# Bioactive fish collagen peptides weaken intestinal inflammation by orienting colonic macrophages phenotype through mannose receptor activation

**DOI:** 10.1007/s00394-021-02787-7

**Published:** 2022-01-08

**Authors:** Mouna Rahabi, Marie Salon, Christelle Bruno-Bonnet, Mélissa Prat, Godefroy Jacquemin, Khaddouj Benmoussa, Mohamad Alaeddine, Mélissa Parny, José Bernad, Bénédicte Bertrand, Yannick Auffret, Pascale Robert-Jolimaître, Laurent Alric, Hélène Authier, Agnès Coste

**Affiliations:** 1 UMR 152 Pharma Dev, Université de Toulouse, IRD, UPS, Toulouse, France; 2 RESTORE Research Center, Université de Toulouse, INSERM, CNRS, EFS, ENVT, Université P. Sabatier, Toulouse, France; 3 Weishardt International, Rond-Point Georges Jolimaître, BP 259, 81305 Graulhet, France; 4 Department of Internal Medicine and Digestive Diseases, Pôle Digestif, CHU Toulouse, Toulouse, France

**Keywords:** Gut inflammation, Innate and adaptive immunity, Collagen, Mannose receptor, Microbiota

## Abstract

**Purpose:**

Particular interest is now given to the potential of dietary supplements as alternative non-pharmacological approaches in intestinal inflammation handling. In this aim, this study evaluates the efficiency of fish collagen peptides, Naticol^®^Gut, on colonic inflammation.

**Methods:**

Wild type and Mannose receptor-deficient in the myeloid lineage C57BL/6 mice were administered with Dextran Sodium Sulfate (DSS), Naticol^®^Gut, DSS, and Naticol^®^Gut or only water for 4 or 8 days. Inflammatory status was evaluated by establishing macroscopic and microscopic scores, by measuring cytokine and calprotectin production by ELISA and the myeloperoxidase activity by chemiluminescence. Colonic macrophages were phenotyped by measuring mRNA levels of specific markers of inflammation and oxidative status. Colonic immune populations and T-cell activation profiles were determined by flow cytometry. Mucosa-associated gut microbiota assessment was undertaken by qPCR. The phenotype of human blood monocytes from inflammatory bowel disease (IBD) subjects was characterized by RT-qPCR and flow cytometry and their oxidative activity by chemiluminescence.

**Results:**

Naticol^®^Gut-treated DSS mice showed attenuated colonic inflammation compared to mice that were only exposed to DSS. Naticol^®^Gut activity was displayed through its ability to orient the polarization of colonic macrophage towards an anti-inflammatory and anti-oxidant phenotype after its recognition by the mannose receptor. Subsequently, Naticol^®^Gut delivery modulated CD4 T cells in favor of a Th2 response and dampened CD8 T-cell activation. This immunomodulation resulted in an intestinal eubiosis. In human monocytes from IBD subjects, the treatment with Naticol^®^Gut also restored an anti-inflammatory and anti-oxidant phenotype.

**Conclusion:**

Naticol^®^Gut acts as a protective agent against colitis appearing as a new functional food and an innovative and complementary approach in gut health.

**Supplementary Information:**

The online version contains supplementary material available at 10.1007/s00394-021-02787-7.

## Introduction

Inflammatory bowel diseases (IBD), mainly represented by Crohn’s disease (CD) and ulcerative colitis (UC), are chronic inflammatory diseases that affect the gastrointestinal tract. Although the etiology of IBD remains largely unclear, it involves a complex interaction between genetics, environmental or microbial factors, and the immune responses [1].

Among innate immune cells, *lamina propria* resident macrophages represent the most abundant mononuclear phagocytic population and are essential for local homeostasis by ensuring harmonious cross-talk between commensal microbiota and the host [2]. These cells, considered as non-inflammatory macrophages, act as scavengers of microbes, assist in the maintenance of regulatory T cells (Treg), and promote epithelial cell renewal through the secretion of interleukin (IL)-10 and transforming growth factor (TGF)-β [3–5]. Thus, under physiological conditions, intestinal macrophages show a permissive phenotype allowing inertia to the commensal microbiota, thereby avoiding inappropriate immune reactions that can lead to continuous inflammation [6]. In IBD pathogenesis, the massive infiltration of activated macrophages majorly contribute to the paracellular leakage within the epithelium disturbing intestinal barrier function by the production of pro-inflammatory mediators such as tumor necrosis factor (TNF)-α, IL-1β, IL-6, IL-12, arachidonic acid (AA) metabolites, and reactive oxygen or nitrogen species [7–9]. Moreover, there is a disruption of the symbiotic relationship with the commensal microbiota causing an overactivation of the mucosal immune system and a dysbiosis. However, whether the dysbiosis is the primary event or a consequence of the immune dysregulation is still unknown [10].

The emergence of IBD is believed to be associated with the environment as suggested by a rapid incidence increase over the past several decades in low-incidence parts of the world. Specifically, the introduction of the “Western diet” has been proposed as an explanation for this increase [11, 12]. Indeed, diet can influence intestinal inflammation by different pathways, (1) altering gut microbiota, (2) affecting gut permeability, or by (3) triggering immune responses by food acting as antigens [13]. Thus, nutrients intake by dietary supplements can modulate the gut inflammatory status. Many approaches use probiotics in intestinal inflammation handling [14], but only a few studies have focused on the use of nutrients such as collagen peptides in this context.

Collagen is the most abundant protein in the human body as it represents one-quarter to one-third of all of the body proteins. Collagen represents the main structural protein that strengthens the tendons, bones, and skin. In mammals, type I and type III collagens account for 75–90% of total collagen [15]. In general, various beneficial effects have been reported on the consumption of collagen hydrolysates, including improvement of joint pain [16], wound healing [17–20], blood pressure [21–23], atherosclerosis [23], and obesity and its metabolic-associated disorders [24]. Intra-articular administration of collagen in the knee of patients with osteoarthritis significantly reduces local inflammation, by increasing Treg and IL-10 and by decreasing, IL-1β thereby alleviating the symptoms inherent to the pathology [25]. Similarly, oral administration of collagen to mice with post-traumatic osteoarthritis inhibits synovial inflammation and induces cartilage regeneration [26]. In metabolic diseases, oral administration of collagen improves metabolic disorders  by directly targeting inflammatory processes [24]. Interestingly, it has also been shown that collagen hydrolysates inhibit zymosan-induced inflammation [27] and that they have a protective effect against gastric ulcers [28, 29], highlighting the significant anti-inflammatory potential of collagen peptides.

The CD280/Endo180/uPARAP (urokinase Plasminogen Activator Receptor-Associated Protein) receptor encoded by the *mrc2* gene [30] as well as the MR (mannose receptor) (CD206/*mrc1*), both belonging to the Mannose Receptor family and expressed by macrophages are involved in the collagen recognition and processing [31]. Indeed, it has been shown, in vivo, that MR specifically expressed by M2-like macrophages is responsible for the recognition and endocytosis of collagen across the fibronectin type II domain by recognizing both native collagen and hydrolyzed collagen peptides [32–36].

An increasing number of studies have been conducted to determine the effects of collagen peptides on inflammation, but until now, there is no evidence on their impact on intestinal inflammation during colitis. Here, we assessed the anti-inflammatory and anti-oxidant potential of Naticol^®^Gut, type I and type III fish collagen peptides, obtained from selected fish skins, in an experimental murine model of DSS-induced colitis. This model is based on a chemical induction of intestinal inflammation with epithelial damages and symptoms that can be assimilated to human ulcerative colitis [37]. Our results demonstrate that Naticol^®^Gut reduces colitis severity in a macrophage-dependent manner. Indeed, following Naticol^®^Gut administration, macrophages exhibit an anti-inflammatory and anti-oxidant phenotype resulting in a decreased Th1/Th2 CD4 T-cell balance and a weakened CD8 T-cell activation. Subsequently, immune system modulation by Naticol^®^Gut administration leads to a maintenance of intestinal eubiosis. Promisingly, the efficiency of Naticol^®^Gut was also validated on human blood monocytes from IBD subjects.

Altogether, our data support the relevance of the development of alternative non-pharmacological approaches using collagen peptides for the management of IBD. These findings also pave the way for further studies in the field of immunomodulation by bioactive nutrients in other pathophysiological contexts.

## Materials and methods

### Mice

Animal work was carried out with Approval No 5412-201051917498658 from the institutional ethics committee (CEEA122) and in accordance with all animal experiments following the principles of animal care and use defined by the European legal and institutional guidelines (2010/63/UE). Eight-week-old C57BL/6 female mice were purchased from *Janvier Labs* (France) for colitis experimentation. At arrival, animals were left for acclimation in the facility for 2 weeks in the conditions described below. Myeloid-specific *MR*^*−/−*^ mice have been described earlier [38] and were also used for bone morrow-derived macrophages generation. The corresponding floxed littermates were used as a control in all experiments. All mice were bred in the same facility and given access to maintenance food (Global Diet, Harlan, France) and water ad libitum. Mice were randomly housed 6 per cage in wire-bottom cages and the facility was temperature-controlled with light/dark cycles of 12 h/12 h. The experimenters were blinded to mice group for animal monitoring.

### Induction of colitis

Mice colonic inflammation was induced by oral administration of Dextran Sodium Sulfate (DSS, MW 36.000–50.000, MP Biomedicals, France) in drinking water at a final concentration of 1.5% (wt/vol) ad libitum from day 1 to day 8 with or without addition of Naticol^®^Gut. Controls received drinking water only. For macrophage polarization kinetics assessment, another mice group received DSS until day 4 then DSS and Naticol^®^Gut until day 8. For Liposomal Clodronate experiments (Xygieia Bioscience, 250 µg per mouse), intraperitoneal (*i.p.*) injections were realized 1 day before DSS protocol and then every 3 days. Animals were monitored daily for weight loss and signs of colitis (diarrhea, bloody stools).

For induction of 2,4,6-trinitrobenzenesulfonic acid (TNBS) colitis, mice were anesthetized and intrarectally injected with 150 mg/kg TNBS (Sigma, France) dissolved in 0.9% NaCl and mixed with an equal volume of ethanol. Control mice received 50% ethanol intrarectally.

After treatment, mice were euthanized by CO_2_ inhalation, and their colons were collected for further analyses.

### Product

The product used in this study is composed of type I and type III fish collagen peptides enzymatically hydrolyzed from fish skins. The product is available under the commercial name of Naticol^®^Gut and provided by Weishardt Group (Graulhet, France). In the supplemented diet, Naticol^®^Gut was added to drinking water at 0.1 g/kg/day. The mean molecular weight of Naticol^®^Gut was evaluated by standardized GME (Gelatine Manufacturers of Europe), method was equal to 2 kDa, and its amino acid composition is presented in supplementary Table 1.

### Sampling procedure

For routine procedure, mice from DSS protocols were sacrificed at day 8 and at day 4 and 8 for kinetics assessment and mice from TNBS protocol, 24 h after TNBS administration. Colons were isolated by cutting from the cecum to the distal end of the rectum, removed under sterile conditions, then weighted and measured for macroscopic and microscopic scores assessment. Colonic immune cells, proteins, inflammatory markers, and microbiota composition were quantified and characterized. Blood was sampled by cardiac blood collection. For calprotectin quantification, blood was left 2 h at room temperature for blood clotting and then centrifuged at 1000*g* for 15 min for serum collection.

### Assessment of colitis severity

Total macroscopic score was assessed as previously described [39]. For histopathological analysis, a portion from distal colon was fixed overnight in 4% paraformaldehyde and embedded in paraffin; sections were stained with hematoxylin and eosin and scored according to Ameho criteria [40].

### Human monocytes

Human subjects’ study was sponsored by the University Hospital of Toulouse for regulated and ethical submission (Clinical trials.gov NCT01990716). Human blood was obtained from IBD patients and collected in BD Vacutainer^®^ CPT™ Mononuclear Cell Preparation Tubes with Sodium Heparin (BD Biosciences). Mononuclear cells were isolated following the manufacturer instructions and monocytes were isolated by adherence to plastic for 2 h in SFM (Gibco) at 37 °C, 5% CO_2_. Monocytes were then plated for reactive oxygen species (ROS) release assessment, and gene and protein expression after addition of Naticol^®^Gut (100 µg/ml) for 6 h.

### ELISA quantification

Serum calprotectin levels were evaluated by performing the Mouse CALP (Calprotectin) ELISA Kit (Elabscience, USA) from mice blood as recommended by the user manual.

Cytokines were quantified from the whole colon mucosae and from culture supernatant of colonic macrophages or BMDM with ELISA kits for TNF-α, IL-1β, IL-6, TGF-β, and IL-10 (BD Biosciences, R&D Systems) according to the manufacturer’s instructions.

### Myeloperoxidase activity (MPO)

MPO activity in colon tissue was measured via chemiluminescence in the presence of 5-amino-2,3-dihydro-1,4-phthalazinedione (100 μM) and hydrogen peroxide (0.1 M) using a luminometer (EnVision, PerkinElmer). Data were normalized by reporting the results to 1 g of colonic tissue. Statistical analysis was performed using the area under the curve expressed in counts × second.

### Isolation of colon immune cells and macrophages

Isolation of murine *lamina propria* mononuclear cells was performed in mice colons, Colons were excised between the distal end of the rectum and the cecum, washed with cold PBS, and cut longitudinally into approximatively 1 cm length pieces. Colon pieces were incubated twice in RPMI 5% FCS, 0.2 mM EDTA for 20 min at 37 °C with slow, regular shaking. Tissue suspensions were passed through a 100 μm nylon membrane after each incubation. Pieces were then consecutively digested three times for 20 min at 37 °C with slow, regular shaking in an enzymatic solution containing collagenase D (0.2 mg/ml, Sigma, France), dispase-II (3 mg/ml, Roche, Mannheim, Germany), and Dnase-I (0.2 mg/ml, Roche) in RPMI 10% FCS. Cells were finally collected by centrifugation and resuspended for further characterization [38, 41],

### Bone marrow-derived macrophages generation

To generate bone marrow-derived macrophages (BMDM), leg bones from *Janvier Labs* wild-type mice and *MR*^+/+^ and *MR*^*−*/*−*^ mice were harvested, cut at each end, and skin and muscles were removed. Bones were then washed with 70% ethanol then PBS. A 25-gauge needle was used to flush out bone marrow with complete DMEM [2 mM l-glutamine, 1% (v/v) penicillin streptomycin solution (Sigma), 10% FBS (Thermo Fisher): cDMEM]. The suspension was then passed through a 40 μM cell strainer (Easy strainRer™ Greiner bio-one), and centrifugated and resuspended in cDMEM. BMDM were matured using M-CSF (30 ng/ml) over 7 days (5% CO_2_, 37 °C) and then primed with lipopolysaccharide (LPS, 10 ng/ml; Sigma, France) overnight before 6 h treatment with Naticol^®^Gut (100 µg/ml).

### RT-qPCR

F4/80 colonic cells were isolated with the anti-F4/80 MicroBeads UltraPure, mouse (Miltenyi Biotec) as recommended by the manufacturer’s instructions. mRNA from F4/80+ cells, from mice colon, BMDM, and from IBD patients’ monocytes was extracted with the EZ-10 Spin Column Total RNA Minipreps Super kit (BioBasic) and reverse transcription of mRNA was performed with the Verso cDNA kit (Thermo Fisher Scientific) following the manufacturer’s recommendations.

RT-qPCR was performed on a LightCycler^®^ 480 system using LightCycler^®^ SYBR Green I Master (Roche Diagnostics). Mouse GAPDH and human 18S ribosomal mRNA were used as the invariant controls in gene expression. Serially diluted samples of pooled cDNA were used as calibration standards in each run for cDNA quantification. Primer sequences are listed in supplementary Table 2 and 3.

### Flow cytometry

Flow cytometry was performed on the dissociated colon cell suspension using a BD LSR Fortessa (BD Bioscience) and analyzed with Diva Software. Viable cells were negative for the staining with Live/Dead Fixable Violet dead cell stain (Lifetechnology). For the evaluation of immune populations, cells were then labeled with the following antibodies: CD45-PerCPVio700, CD11b-FITC, F4/80-APC, CD3-PE, CD8-VioGreen, CD4-VioBrightFITC, and CD25-PEVio770 (Miltenyi Biotec).

T lymphocyte profiling studies were performed by intracellular staining with the inside Stain Kit and the following antibodies: IFN-γ-FITC, IL-2-PE, and TNF-α-APC for the Th1 profile and IL-10-FITC, IL-4-PE, and IL-5-APC for the Th2 profile and Treg were evaluated with Foxp3-Vio667 as recommended by the manufacturer (Miltenyi Biotec).

Appropriate fluorochrome-matched isotype antibodies were used to determine nonspecific background staining. All stainings were performed on 100 µl of PBS without calcium and magnesium and 1% heat-inactivated FCS.

A multiplex bead-based immunoassay was used (Biolegend, LEGENDPlex™ Mix and Match System) for cytokine and Arginase-1 titration from IBD patients’ monocytes.

### ROS release assessment

The oxygen-dependent respiratory burst (ROS production) was assessed on C57/BL6 colonic macrophages from DSS mice and on monocytes from IBD patients treated or not with Naticol^®^Gut. Cells were incubated in the presence of 5-amino-2,3-dihydro-1,4-phthalazinedione (luminol, Sigma-Aldrich) and challenged with 12-O-tetradecanoyl-phorbol-13-acetate (TPA, 100 µM, Sigma-Aldrich). ROS production was measured by chemiluminescence by continuously monitoring for 1 h using a thermostatically (37 °C) controlled luminometer (Envision; Perkin Elmer). Statistical analysis was performed using the area under the curve expressed in counts × seconds.

### Microbiota characterization

DNA from mice colons was purified using High Pure PCR Template preparation kit (Roche Mannheim, Germany). To evaluate bacterial and fungal populations associated with the mucosae, semi-quantitative PCR was performed on the extracted DNA using primers listed in supplementary Table 4. Relative quantity was normalized to the amount of β-actin, total bacteria, or fungi as well as to their respective phylum, family, or genus. LightCycler FastStart DNA SYBR Green I kit was used for amplicon detection and PCR performed on a LightCycler^®^ 480 system (Roche Diagnostics).

For in vitro microbiology studies, bacterial and fungal species listed in supplementary Table 5 were purchased from DSMZ (Germany) and ATCC and cultured in their specific media. To determine whether Naticol^®^Gut has an antimicrobial or a proliferative activity, a specific concentration of each bacteria and yeast was then transferred into a 96-well microtitration plate on a minimum broth medium with addition of several twofold dilutions of Naticol^®^Gut. After 36 h, the optical density (OD) at 600 nm was measured to determine bacterial and fungal concentrations. OD values were reported to the calibration standard curve of the respective specie.

### Quantification and statistical analysis

Data were expressed as mean ± SEM and are representative of three independent experiments. For each experiment, data were subjected to one-way analysis of variance followed by multiple comparisons of means using the Bonferroni–Dunnett method. *P* value < 0.05 was defined as the level of statistical significance.

## Results

### In vivo Naticol^®^Gut administration attenuates colonic inflammation during colitis

Dextran Sodium Sulfate (DSS)-induced colitis protocol represents the most widely used model of colitis. This chemical colitogen has anticoagulant properties and acts by damaging the epithelial monolayer of the large intestine resulting in the propagation of pro-inflammatory content into underlying tissue [42]. Hence, this model is useful for studying the implication of the innate immune system and gut microbiota in the development of colitis.

**Fig. 1:**
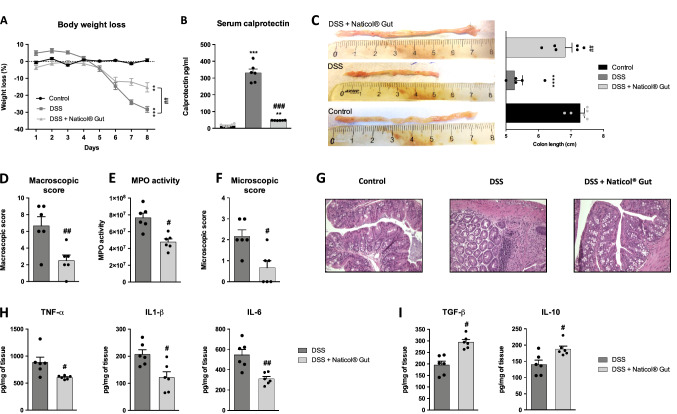
In vivo Naticol^®^Gut administration dampens colonic inflammation during DSS-induced colitis. **A** Body weight loss, **B** serum calprotectin, **C** colon length, **D** macroscopic scores, **E** MPO activity, **F** microscopic scores, **G** H&E staining of representative cross-sections of distal colon, and **H**, **I** TNF-α, IL-6, IL-1β, TGF-β, and IL-10 protein levels were determined at day 8  in the colons of control or DSS-exposed mice treated or not with Naticol^®^Gut (*n* = 6 per group). **p ≤ 0.01, ***p ≤ 0.005, ****p ≤ 0.001 compared to control mice (DSS-unexposed mice). ^#^*p* ≤ 0.05, ^##^*p* ≤ 0.01, ^###^*p* ≤ 0.005 compared to DSS-exposed mice

The administration of Naticol^®^Gut to DSS-treated mice significantly decreased the body weight loss compared to DSS-treated mice without Naticol^®^Gut administration (Fig. 1a). Moreover, serum calprotectin level, which positively correlates with the inflammatory status and thereby with the severity of the disease, is strongly decreased in Naticol^®^Gut-treated DSS mice (Fig. 1b).

The macroscopic study of the colon indicated a reduced colon shortening and a lower macroscopic score in Naticol^®^Gut-treated DSS mice (Fig. 1c, d). Consistent with these findings, Naticol^®^Gut-treated DSS mice displayed a lower degree of colonic inflammation as reflected by a strong decrease of the MPO activity (Fig. 1e), a lower microscopic score (Fig. 1f) and less immune cells infiltrate, glandular ulceration, crypts loss, and epithelial erosion on histological section of the distal colon (Fig. 1g).

The attenuated colonic inflammation in Naticol^®^Gut-treated DSS mice was associated with lower levels of TNF-α, IL-1β, and IL-6 pro-inflammatory cytokines in the colonic tissue (Fig. 1h) and higher TGF-β and IL-10 anti-inflammatory cytokines secretion (Fig. 1i). The anti-inflammatory activity of Naticol^®^Gut was further supported in a TNBS-induced experimental colitis murine model (Supplementary Fig. 1). Altogether, these data highlighted Naticol^®^Gut as a protective agent against colonic inflammation during colitis.

### Naticol^®^Gut administration orients colonic macrophages towards an anti-inflammatory and anti-oxidant phenotype during colitis

**Fig. 2:**
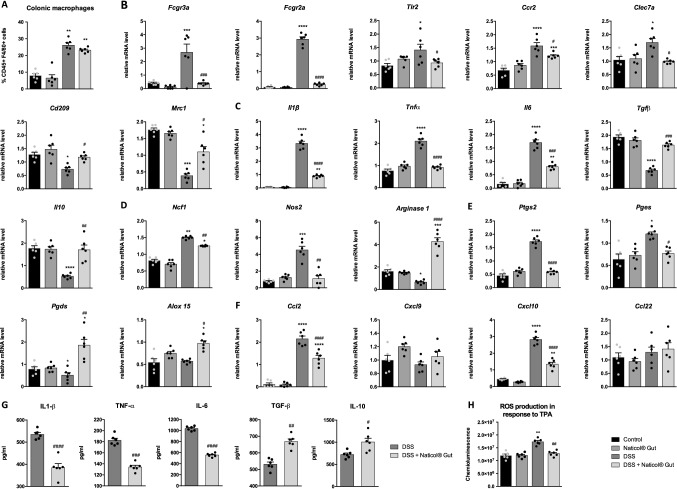
Naticol^®^Gut administration orients colonic macrophages towards an anti-inflammatory and anti-oxidant phenotype during colitis. **A** Percentage of macrophages in the colon of control or DSS-exposed mice treated or not with Naticol^®^Gut.. Cells were isolated by enzymatic digestion of colons, and among viable cells, the macrophages were identified as CD45^+^ CD11b^+^ and F4/80^+^ by flow cytometry. **p ≤ 0.01, compared to control mice (DSS-unexposed mice). **B**–**F** Gene expression analysis of inflammatory and oxidative stress markers in colonic macrophages from control or DSS-exposed mice treated or not with Naticol^®^Gut using qRT-PCR. Results represent relative mRNA levels. Data are representative of three independent experiments (*n* = 6 per group). *p ≤ 0.05, **p ≤ 0.01, ***p ≤ 0.005, ****p ≤ 0.001 compared to control mice (DSS-unexposed mice). ^#^*p* ≤ 0.05, ^##^*p* ≤ 0.01, ^###^*p* ≤ 0.005, ^####^*p* ≤ 0.001 DSS-exposed mice compared to DSS-exposed mice treated with Naticol^®^Gut. **G** IL-1β, TNF-α, IL-6, TGF-β, and IL-10 release by colonic macrophages from DSS-exposed mice treated or not with Naticol^®^Gut. ^#^*p* ≤ 0.05, ^##^*p* ≤ 0.01, ^###^*p* ≤ 0.005, ^####^*p* ≤ 0.001 compared to DSS-exposed mice not treated with Naticol^®^Gut. **H** ROS production by colonic macrophages from DSS-unexposed mice treated or not with Naticol^®^Gut and DSS-exposed mice treated or not with Naticol^®^Gut upon (TPA) challenge. **p ≤ 0.01, compared to control mice (DSS-unexposed mice). ^##^*p* ≤ 0.01, DSS-exposed mice compared to DSS-exposed mice treated with Naticol^®^Gut

As macrophages are known to play a key role in the pathophysiology of IBD, we first evaluated their mucosal infiltration in untreated and Naticol^®^Gut-treated DSS mice. Although DSS induced a marked increase of the percentage of macrophages (CD45^+^, CD11b^+^, F4/80^+^ cells) in the colon, the administration of Naticol^®^Gut did not impact their proportion (Fig. 2a).

Since inflammation in colitis is closely dependent on the polarization state of macrophages [2], we further explored the expression of specific pro-inflammatory and anti-inflammatory markers in macrophages isolated from colon (Fig. 2b). Overall, DSS increased the expression of surface receptor characteristics of inflammatory phenotype, such as *Fcgr3a (*CD16), *Fcgr2a* (CD32), *Tlr2, Ccr2*, and *Clec7a* (dectin-1) in colonic macrophages. This was mirrored by a downregulation of *Cd209* (SIGNR1) and *Mrc1* (Mannose Receptor) anti-inflammatory markers. Interestingly, while the mRNA levels of *Cd209* and *Mrc1* were up-regulated in colonic macrophages from Naticol^®^Gut-treated DSS mice, the expression of *Fcgr3a*, *Fcgr2a*, *Tlr2, Ccr2*, and *Clec7a* was down-regulated (Fig. 2b). Similarly, the induction of mRNA and protein levels of IL-1β, TNF-α, and IL-6 pro-inflammatory cytokines in colonic macrophages was strongly reduced in macrophages from Naticol^®^Gut-treated DSS mice (Fig. 2c–g). These findings were supported by the reciprocal increase of TGF-β and IL-10 anti-inflammatory cytokine mRNA and protein expression in colonic macrophages from Naticol^®^Gut-treated DSS mice (Fig. 2c–g).

Consistent with reduced pro-inflammatory markers in macrophages from Naticol^®^Gut-treated DSS mice, Naticol^®^Gut administration orients the balance between *Nos2* (inducible nitric oxide synthase) and *Arginase1* toward *Arginase1* expression (Fig. 2d). This was reinforced by the strong decrease of enzymes involved in pro-inflammatory eicosanoid synthesis, such as *Ptgs2* (Cyclooxygenase-2) and *Pges* (Prostaglandin E synthase), and the reciprocal increase of *Pgds* (Prostaglandin D synthase) and *Alox15* (12/15-lipoxygenase), implicated in the anti-inflammatory bioactive lipid production (Fig. 2e).

Interestingly, the administration of Naticol^®^Gut inhibited the expression of *Ccl2* and *Cxcl10* pro-inflammatory chemokines, which were strongly induced upon DSS treatment (Fig. 2f). Altogether, these results demonstrate that the attenuation of colonic inflammation by Naticol^®^Gut administration is correlated to its capacity to orient the phenotype of macrophages toward an anti-inflammatory phenotype.

Regarding the oxidative stress status of colonic macrophages, we demonstrated that the increased capacity of colonic macrophages from DSS mice to produce ROS upon TPA stimulation is suppressed in macrophages from Naticol^®^Gut-treated DSS mice (Fig. 2h). In line, the mRNA expression of *Ncf1* (p47^phox^), a cytosolic subunit of the NADPH oxidase complex whose activation is essential to ROS release, was down-regulated in macrophages from Naticol^®^Gut-treated DSS mice (Fig. 2d). Thus, besides its anti-inflammatory potential, Naticol^®^Gut also decreases the pro-oxidative activity of colonic macrophages during colitis.

To determine whether Naticol^®^Gut is capable to directly modulate macrophage polarization, the anti-inflammatory and anti-oxidant potential of Naticol^®^Gut was evaluated on LPS-activated bone marrow-derived macrophages (BMDM) (Supplementary Fig. 2). Interestingly, upon Naticol^®^Gut stimulation, the induction by LPS treatment of *Fcgr3a*, *Tlr2*, *Il1β*, *Tnfα*, *Il6*, *Ptgs2, Pges, Ccl2, Cxcl10, Ncf1* and *Nos2*, pro-inflammatory and pro-oxidant markers in BMDM were strongly diminished, whereas the decrease of *Mrc1*, *Tgfβ, Il10*, and *Arginase1* mRNA levels was significantly reduced, supporting that the anti-inflammatory and anti-oxidant potential of Naticol^®^Gut is directly exerted on macrophages. Altogether these results reveal that when Naticol^®^Gut is concomitantly administered with DSS, it prevents the orientation of macrophage polarization towards a pro-inflammatory phenotype by maintaining their differentiation closer to the physiological state.

**Fig. 3:**
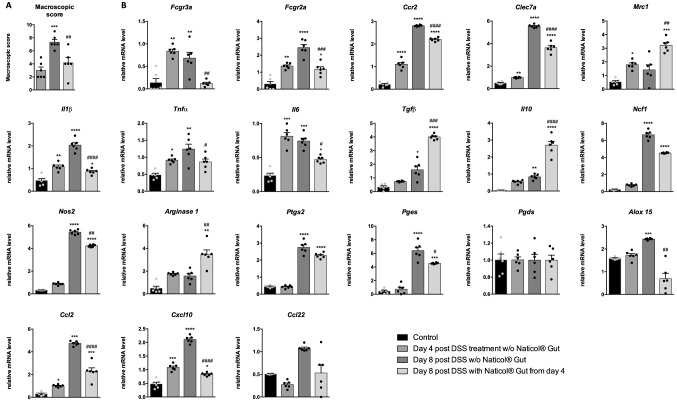
Naticol^®^Gut reverses pro-inflammatory and pro-oxidant colonic macrophages towards an anti-inflammatory and anti-oxidant phenotype during colitis. **A** Macroscopic scores, **B** gene expression analysis of inflammatory and oxidative stress markers in colonic macrophages from DSS-unexposed mice (control), mice exposed to DSS during 4 days or 8 days and mice exposed to DSS during 8 days treated with Naticol^®^Gut from day 4 post-DSS administration (*n* = 6 per group). Results represent relative mRNA levels. *p ≤ 0.05, **p ≤ 0.01, ***p ≤ 0.005, ****p ≤ 0.001 compared to control mice (DSS-unexposed mice). ^#^*p* ≤ 0.05, ^##^*p* ≤ 0.01, ^###^*p* ≤ 0.005, ^####^*p* ≤ 0.001 untreated DSS-exposed mice during 8 days compared to DSS-exposed mice treated with Naticol^®^Gut from day 4 post-DSS administration

To evidence whether Naticol^®^Gut can also reverse macrophage pro-inflammatory phenotype after the colitis development, we initiated the Naticol^®^Gut treatment only 4 days after DSS administration and evaluated colonic macroscopic scores and macrophages’ polarization at day 8 (Fig. 3). As expected, colonic inflammation increased from day 4 to day 8 after DSS treatment and was attenuated when Naticol^®^Gut was administered from day 4 (Fig. 3A), Moreover, DSS administration promoted the expression of pro-inflammatory and pro-oxidant markers in colonic macrophages (*Fcgr3a, Fcgr2a*, *Ccr2*, *Clec7a*, *Il1β*, *Tnfα*, *Il6*, *Ptgs2, Pges, Ccl2, Cxcl10, Ncf1*, and *Nos2*) from day 4 post-DSS administration which were amplified at day 8 (Fig. 3b). Interestingly, the treatment with Naticol^®^Gut from day 4 post-DSS administration reduced significantly their expression, suggesting that Naticol^®^Gut can reverse the inflammatory phenotype of macrophages. Correspondingly, Naticol^®^Gut up-regulated mRNA expression of *Mrc1*, *Tgfβ, Il10*, and *Arginase1* anti-inflammatory markers (Fig. 3b).

Thus, the administration of Naticol^®^Gut to mice can prevent the switch of colonic macrophage towards a pro-inflammatory and pro-oxidant phenotype as it can also reverse their pro-inflammatory and pro-oxidant profile towards an anti-inflammatory and anti-oxidant status.

### Naticol^®^Gut administration modulates Th1/Th2 balance of CD4 T cells in favor of a Th2 profile and dampens CD8 cytotoxic T-cell activation during colitis

**Fig. 4:**
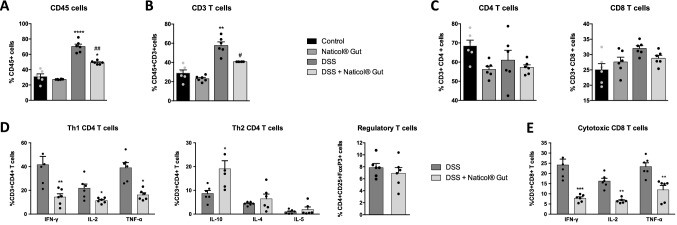
Naticol^®^Gut administration orients colonic CD4 and CD8 T cells towards an anti-inflammatory status during colitis. Percentage of leukocytes (**A**), T cells (**B**), and CD4 and CD8 T cells (**C**) in the colon of DSS-unexposed mice treated or not (control) with Naticol^®^Gut and of DSS-exposed mice treated or not with Naticol^®^Gut (*n* = 6 per group). Cells were isolated by enzymatic digestion of colons and among viable cells, the leukocytes, T cells and CD4+ and CD8+ T cells were, respectively, identified as CD45^+^, CD45^+^ CD3^+^ and CD45^+^ CD3^+^ CD4+ or CD8+ by flow cytometry. *p ≤ 0.05, **p ≤ 0.01, ****p ≤ 0.001 compared to control mice (DSS-unexposed mice). ^#^*p* ≤ 0.05, ^##^*p* ≤ 0.01 DSS-exposed mice compared to DSS-exposed mice treated with Naticol^®^Gut. **D** Percentage of Th1, Th2, and Treg CD4+ T cells in the colon from DSS-exposed mice treated or not with Naticol^®^Gut. To evaluate Th1/Th2 activation of CD4+ T cells, their intracellular production of IFN-γ, IL-2, TNF-α, IL-10, IL-4, and IL-5 was assessed by flow cytometry. Regulatory T cells were identified as CD4+ CD25+ FoxP3 by flow cytometry. *p ≤ 0.05, **p ≤ 0.01 compared to DSS-exposed mice. **E** Percentage of cytotoxic CD8 T cells in the colon from DSS-exposed mice treated or not with Naticol^®^Gut. To evaluate CD8 T-cell activation, their intracellular production of IFN-γ, IL-2, and TNF-α was assessed by flow cytometry. **p ≤ 0.01, ***p ≤ 0.005 compared to DSS-exposed mice

To establish whether the orientation of colonic macrophages upon Naticol^®^Gut treatment impacts the recruitment and the activation of adaptive immune cells, we evaluated the proportion and the activation state of colonic CD4+ and CD8+ T cells (Fig. 4). As expected, DSS treatment increased the proportion of CD45+ immune cells into the colon, whereas the administration of Naticol^®^Gut reduced this percentage (Fig. 4a). This was mainly due to T-cell variation as demonstrated by a decrease in the percentage of CD3+ T cells (Fig. 4b) and the unchanged proportion of macrophages (F4/80+ cells) in the colon (Fig. 2a).

Although Naticol^®^Gut administration significantly decreased CD3+ T-cell infiltration (Fig. 4b), it did not alter colonic CD4+ and CD8+ T-cell proportions (Fig. 4c). However, we demonstrated that Naticol^®^Gut decreased the percentage of CD4+ T cells producing IFN-ɣ, IL-2 and TNF-α in colon (Fig. 4d). Consistently, Naticol^®^Gut expanded the percentage of CD4+ T cells secreting IL-10, IL-4, and IL-5, demonstrating that Naticol^®^Gut orients CD4+ T cells toward a Th2 profile (Fig. 4d). Furthermore, the proportion of CD4+ CD25+ Foxp3+ Treg in the colon was not modified upon Naticol^®^Gut treatment (Fig. 4d). Considering CD8+ T cells, Naticol^®^Gut diminished the proportion of CD8+ T cells producing IFN-ɣ, IL-2 and TNF-α in the colon (Fig. 4e), supporting Naticol^®^Gut as a component able to attenuate Th1 functional properties of CD4 T cells and CD8 T-cell cytotoxicity.

Altogether, these data demonstrate that Naticol^®^Gut strengthens Th2 CD4 T-cell population and diminishes the activation of CD8 T cells in colon during experimental DSS-induced colitis.

### Naticol^®^Gut administration indirectly impacts gut microbiota by promoting the development of probiotic species and limiting pathobiontic microorganisms during colitis

**Fig. 5:**
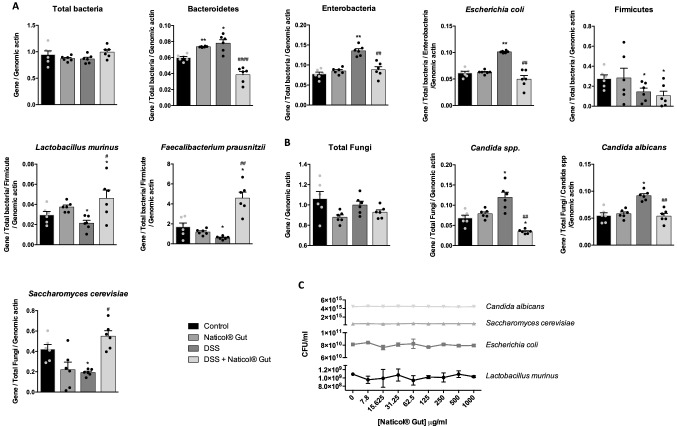
Naticol^®^Gut administration results in an intestinal eubiosis characterized by a limitation in the development of pathobiontic species for the benefit of probiotic species. **A**, **B** Intestinal mucosae-attached microbiota of DSS-unexposed mice treated or not (control) with Naticol^®^Gut and of DSS-exposed mice treated or not with Naticol^®^Gut (*n* = 6 per group). Values were normalized to total bacteria or total fungi and host β-actin. *E. coli* was normalized to total bacteria, Enterobacteria, and host β-actin. *L. murinus and F. prausnitzii* were normalized to total bacteria, Firmicutes, and host β-actin. *C. albicans was* normalized to total Fungi, *Candida* spp. and host β-actin. *p ≤ 0.05, **p ≤ 0.01 compared to control mice (DSS-unexposed mice). ^#^*p* ≤ 0.05, ^##^*p* ≤ 0.01, ^####^*p* ≤ 0.001 DSS-exposed mice compared to DSS-exposed mice treated with Naticol^®^Gut. **C** In vitro culture of selected bacteria and yeasts on minimum broth medium with addition of several twofold dilutions of Naticol^®^Gut. After 36 h, the OD_600nm_ was measured and reported to the calibration standard curve of the respective specie

Given that gut microbiota represents one of the most incriminated components in IBD development, we evaluated the mucosae-associated fungal and bacterial colonization. Although DSS treatment did not change the mucosae total bacteria content, it increased the burden of Bacteroïdetes, Enterobacteria, and its pathobiontic specie *Escherichia coli* (Fig. 5a). Interestingly, Naticol^®^Gut administration decreased significantly the content of these Phyla, typically up-regulated under colonic inflammation [43, 44]. Consistently, even if Naticol^®^Gut administration did not impact the load of Firmicutes, it increased the burden of *Lactobacillus murinus,* a probiotic genus ref, and *Faecalibacterium prausnitzii*, a crucial bacterium in intestinal mucosae health [45] (Fig. 5a).

We then evaluated the fungal load associated with the mucosae in DSS mice. Although the total fungal load remained unchanged, DSS led to the typical unbalance between *Candida albicans* and *Saccharomyces cerevisiae*, with an overrepresentation of *Candida spp.* genus, particularly *C. albicans,* and a deprivation of the *S. cerevisiae* probiotic yeast (Fig. 5b). Interestingly, the administration of Naticol^®^Gut restored *C. albicans* and *S. cerevisiae* classical proportions in the colon with a predominant *S. cerevisiae* load. Thus, in Naticol^®^Gut-treated DSS mice, the attenuated severity of colitis was associated both with an increase in protective bacteria and fungi in the colon and with a reduced pathobiontic bacteria and fungi colonization.

We next assessed whether the effect of Naticol^®^Gut in microbiota composition was directly exerted on the microorganism growth or through its effect on mucosal immune system. The in vitro culture of selected bacteria and yeasts on minimal media showed that the bacterial and fungal growths were similar with or without Naticol^®^Gut (Fig. 5c). Altogether, these data demonstrate that Naticol^®^Gut has no direct effect in promoting or inhibiting microorganism development and suggest that the microbiota modulation by Naticol^®^Gut is dependent on its impact on immune system.

### Naticol^®^Gut treatment weakens colonic inflammation and the inflammatory status of colonic CD4 and CD8 T cells through a mechanism dependent on macrophages

**Fig. 6:**
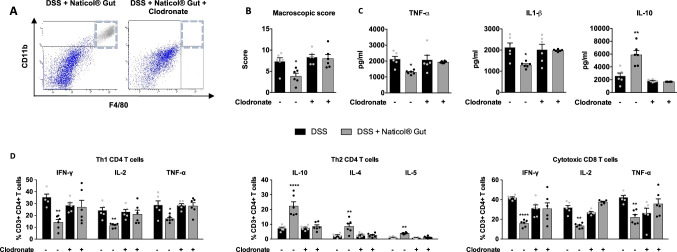
The impact of Naticol^®^Gut administration on colonic inflammation and CD4 and CD8 T-cell activation depends on macrophages. **A** Representative flow cytometry dot plot of macrophages (CD45^+^, CD11b^+^, F4/80^+^) in the colon from DSS-exposed mice treated with Naticol^®^Gut injected or not with clodronate. **B** Macroscopic scores determined at 8 days on DSS-exposed mice treated or not with Naticol^®^Gut and injected or not with clodronate. *p ≤ 0.01 compared to the respective DSS-exposed mice. **C** TNF-α, IL-1β, and IL-10 protein levels in colon from DSS-exposed mice treated or not with Naticol^®^Gut and injected or not with clodronate. *p ≤ 0.01, **p ≤ 0.01 compared to the respective DSS-exposed mice. **D** Percentage of Th1 CD4 T cells and cytotoxic CD8 T cells in the colon from DSS-exposed mice treated or not with Naticol^®^Gut and injected or not with clodronate. To evaluate CD4 and CD8 T-cell activation, their intracellular production of IFN-γ, IL-2, and TNF-α was assessed by flow cytometry. *p ≤ 0.05, **p ≤ 0.01, ****p ≤ 0.001 compared to the respective DSS-exposed mice (*n* = 6 per group)

To further dissect how Naticol^®^Gut attenuates colonic inflammation, we assessed the colonic inflammatory status in DSS mice selectively depleted in macrophages by clodronate-containing liposomes *i.p.* administration (Fig. 6a). Although Naticol^®^Gut reduced significantly colonic inflammation in DSS-treated mice, as demonstrated by a lower macroscopic score and TNF-α and IL-1β production in colon (Fig. 6b, c), it failed to do so in DSS mice depleted in macrophages. Consistently, the higher IL-10 secretion in colon from Naticol^®^Gut-treated DSS mice was abolished when macrophages were depleted, thus establishing the critical involvement of macrophages in the anti-inflammatory activity of Naticol^®^Gut.

To investigate whether the impact of Naticol^®^Gut in the activation of CD4 and CD8 T cell is dependent on its impact on macrophage polarization, we evaluated the proportion of Th1 and Th2 CD4+ T cells and cytotoxic CD8 T cells in colon from Naticol^®^Gut-treated DSS mice selectively depleted in macrophages (Fig. 6d). The capacity of Naticol^®^Gut to decrease the percentage of CD4+ and CD8+ T cells producing IFN-ɣ, IL-2, and TNF-α and to increase the percentage of CD4+ secreting IL-10, IL-4, and IL-5 in colon was lost upon clodronate administration, supporting that Naticol^®^Gut modulates Th1/Th2 balance of CD4 T cells in favor of a Th2 profile and dampens CD8 cytotoxic T cell through its impact on macrophage polarization.

### MR expressed on macrophages is a key target of Naticol^®^Gut to weaken colitis

**Fig. 7:**
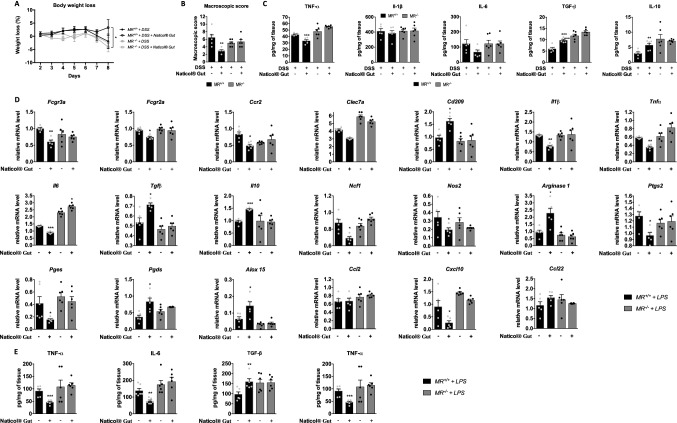
Naticol^®^Gut orients macrophage polarization through mannose receptor. **A** Body weight loss. **B** Macroscopic scores. *p ≤ 0.05, **p ≤ 0.01 as compared DSS-exposed mice compared to the relative DSS-exposed mice treated with Naticol^®^Gut. **C** TNF-α, IL-1β, IL-6, TGF-β, and IL-10 protein levels were determined at day 8 in the colons of DSS-exposed *MR*^+*/*+^ and *MR*^*−/−*^ mice treated or not with Naticol^®^Gut. Results are represented as fold inductions of the protein levels in colon from DSS-exposed mice treated with Naticol^®^Gut in comparison to the protein levels in colon from DSS-exposed mice *p ≤ 0.05, **p ≤ 0.01, ***p ≤ 0.005, ****p ≤ 0.001. **D** Gene expression analysis of inflammatory and oxidative stress markers in bone marrow-derived macrophages (BMDM) from *MR*^+*/*+^ and *MR*^*−/−*^ mice primed with LPS and stimulated or not with Naticol^®^Gut using qRT-PCR. Results represent relative mRNA levels. **E** TNF-α, IL-6, TGF-β, and IL-10 protein levels were determined from the supernatant of BMDM from *MR*^+*/*+^ and *MR*^*−/−*^ mice primed with LPS and stimulated or not with Naticol^®^Gut *p ≤ 0.05, **p ≤ 0.01, ***p ≤ 0.005, ****p ≤ 0.001 compared to the respective LPS-primed BMDM (*n* = 6 per group)

As the mannose receptor (MR) is the most described collagen peptide receptor, we investigated its role in the orientation of the macrophage anti-inflammatory phenotype induced by Naticol^®^Gut treatment. Therefore, we assessed the anti-inflammatory potential of Naticol^®^Gut in mice selectively deleted for the mannose receptor (*MR*^*−/−*^) in the myeloid lineage. Interestingly, the administration of Naticol^®^Gut to DSS-treated *MR*^*−/−*^ mice failed to reduce the body weight loss compared to DSS-treated *MR*^+*/*+^ mice in which Naticol^®^Gut decreased the body weight loss (Fig. 7a). Likewise, in DSS-treated *MR*^*−/−*^ mice, the Naticol^®^Gut lost its capacity to diminish the macroscopic score, highlighting the importance of the MR in the anti-inflammatory effect of Naticol^®^Gut (Fig. 7b). This was associated with reduced inhibition of TNF-α, IL-1β, and IL-6 pro-inflammatory cytokine release by Naticol^®^Gut and an impaired induction of TGF-β and IL-10 anti-inflammatory cytokine release in colon (Fig. 7c).

To further investigate the role of the MR of macrophages, we assessed macrophage polarization by Naticol^®^Gut in LPS-activated BMDM selectively deleted for mannose receptor (*MR*^*−/−*^). Interestingly, the decrease of the expression of *Fcgr3a*, *Fcgr2a*, *Ccr2*, and *Clec7a*, typical pro-inflammatory macrophage surface receptors and the increase of *Cd209*, characteristic of anti-inflammatory phenotype, induced by Naticol^®^Gut treatment in *MR*^+*/*+^ macrophages were suppressed in *MR*^*−/−*^ macrophages. Moreover, the reduction of pro-inflammatory and pro-oxidant mediators by Naticol^®^Gut in LPS-activated macrophages, such as *Il1β, Tnfα, Il6, Ncf1*, *Nos2*, *Ptgs2, Pges*, and *Cxcl10,* was abolished in *MR*^*−/−*^ macrophages (Fig. 7d). Concomitantly, the increase of *Tgfβ, Il10, Arginase1, Pgds*, and *Alox15* anti-inflammatory and anti-oxidant markers in LPS-activated macrophages by Naticol^®^Gut treatment is strongly diminished in *MR*^*−/−*^ macrophages. Consistently, the reduction of IL-6 and TNF-α release and the induction of IL-10 and TGF-β by Naticol^®^Gut were abolished in *MR*^*−/−*^ macrophages (Fig. 7e).

Altogether, these results demonstrate that the deficiency of the MR in macrophages abrogates the anti-inflammatory and anti-oxidant activities of Naticol^®^Gut on macrophages, identifying MR on macrophages as a critical target of Naticol^®^Gut to dampen colitis through the orientation of macrophages toward a protective phenotype.

### Naticol^®^Gut stimulation of human blood monocytes from IBD patients orients their polarization through an anti-inflammatory and anti-oxidant phenotype

**Fig. 8:**
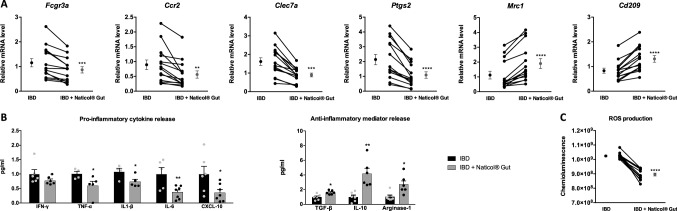
Naticol^®^Gut stimulation promotes anti-inflammatory and anti-oxidant phenotype of human blood monocytes from IBD patients. **A** Gene expression analysis of *Fgcr3a, Ccr2, Clec7a, Ptgs2, Mrc1*, and *Cd209* and in human blood monocytes from IBD subjects stimulated or not with Naticol^®^Gut (*n* = 14) using qRT-PCR. **p ≤ 0.01, ***p ≤ 0.005, ****p ≤ 0.001 compared to the untreated condition. **B** IFN-γ, TNF-α, IL-1β, IL-6, CXCL-10, TGF-β, IL-10, and Arginase-1 release by human blood monocytes from IBD subjects stimulated or not with Naticol^®^Gut using flow cytometry. *p ≤ 0.05, **p ≤ 0.01 compared to the untreated condition. **C** ROS production by human blood monocytes from IBD subjects stimulated or not with Naticol^®^Gut. ****p ≤ 0.001 compared to the untreated condition

To investigate whether the effect of Naticol^®^Gut can be extended to humans, we characterized the impact of Naticol^®^Gut on human blood monocytes from IBD patients which exhibit a strong pro-inflammatory profile. Naticol^®^Gut stimulation of these cells decreased mRNA levels of *Fcgr3a, Ccr2, Clec7a*, and *Ptgs2* pro-inflammatory markers (Fig. 8a). This was mirrored by an up regulation of *Mrc1* and *Cd209* expression (Fig. 8a). Consistently, Naticol^®^Gut also significantly reduced the secretion of IFN-γ, TNF-α, IL-1β, IL-6, and CXCL-10 pro-inflammatory mediators in human blood monocytes from IBD patients (Fig. 8b). Concomitantly, they produced higher levels of TGF-β, IL-10 and Arginase-1 anti-inflammatory mediators (Fig. 8b). In addition to its anti-inflammatory activity, Naticol^®^Gut also diminished ROS release by human blood monocytes from IBD patients upon TPA stimulation (Fig. 8c).

Altogether, these data confirmed that Naticol^®^Gut displays similar contributions in the restoration of anti-inflammatory and anti-oxidant phenotype in human monocytes from IBD subjects, further supporting our results obtained in mouse model.

## Discussion

The use of nutritional supplements is an emerging concept in the management of inflammatory pathologies [14]. In this context, we report here that in vivo administration of natural bioactive type I and type III fish collagen peptides (Naticol^®^Gut) decreased the susceptibility to colitis with a significant reduction in colonic inflammatory parameters, highlighting Naticol^®^Gut as a protective agent against colonic inflammation during DSS-induced colitis. The anti-inflammatory activity of collagen was supported by previous data showing that the administration of collagen in the knee of patients with osteoarthritis significantly reduces local inflammation [25] and that oral administration of collagen in mice with post-traumatic osteoarthritis inhibits synovial inflammation and induces cartilage regeneration [26]. In line with this, recent studies on metabolism have revealed that the beneficial effects of type I and III fish collagen peptides obtained from the same fish skin species are exerted through targeting inflammatory processes [24].

In this study, the depletion of macrophages by the administration of clodronate-containing liposomes abolished the protective effect of Naticol^®^Gut, thus establishing the critical involvement of macrophages in its gut anti-inflammatory activity. In agreement with an attenuated inflammation in Naticol^®^Gut-treated DSS mice, the treatment prevents the switch of colonic macrophage towards a pro-inflammatory status. Indeed, the induction of several inflammation-related genes in colonic macrophages from mice with colitis, including pro-inflammatory cytokines, chemokines, and enzymes involved in pro-inflammatory eicosanoid synthesis, was significantly impaired in macrophages from mice with colitis treated with Naticol^®^Gut. This is supported by reports, showing that type I and III fish collagen peptides decrease the expression of IL-6 and IL-1β in isolated adipocytes [24] and that macrophage collagen receptor signaling is linked to the inhibition of pro-inflammatory macrophage differentiation and altered nitric oxide (NO) and reactive oxygen species (ROS) production in a lung fibrosis context [46].

Interestingly, in addition to its preventive activity on the development of colitis and the acquisition of macrophage pro-inflammatory phenotype, Naticol^®^Gut is also capable of reversing the pro-inflammatory phenotype associated with colitis towards an anti-inflammatory and pro-resolutive profile.

We report here that the anti-inflammatory potential of Naticol^®^Gut during colitis is mediated by the mannose receptor (MR) on macrophages as in mice with *MR*-deficient macrophages, Naticol^®^Gut treatment loses its ability to attenuate colitis and to orient macrophages toward an anti-inflammatory phenotype. The lack of Naticol^®^Gut anti-inflammatory activity in MR-deficient macrophages is supported by our previous study evidencing  a protective role of MR in colonic inflammation [38]. In agreement with our data identifying MR on macrophages as a critical target of Naticol^®^Gut, numerous studies have already demonstrated the involvement of MR (CD206/*mrc1*) in collagen recognition, endocytosis, and processing of both native and hydrolyzed collagen [31–36].

Moreover, our findings showing that Naticol^®^Gut stimulation of human blood monocytes from IBD subjects decreased the expression of pro-inflammatory markers, and concomitantly, positively regulated anti-inflammatory markers, provide evidence that Naticol^®^Gut can reverse monocyte phenotype toward an anti-inflammatory status in human. The fact that food-based collagen peptides are detectable in the human peripheral blood after oral ingestion of collagen peptides supports the relevance of Naticol^®^Gut intake to attenuate the inflammatory status of blood monocytes from IBD patients [47]. In addition, besides its anti-inflammatory potential, Naticol^®^Gut also decreases the pro-oxidative activity of colonic macrophages from mice with colitis and of human monocytes from IBD subjects. This observation was consistent with studies, showing that collagen hydrolysates can increase superoxide dismutase, glutathione reductase, and peroxidase activities and can also inhibit ROS production in several cell lines, as mice embryonic fibroblast, hepatocytes, and macrophages [48–50]. Altogether, these results support the potential use of Naticol^®^Gut as a natural anti-inflammatory and anti-oxidant agent to modulate the phenotype of macrophages, which play a key role in the control of intestinal homeostasis [8].

Consistent with the inhibition of *Cxcl10* expression in colonic macrophages upon Naticol^®^Gut treatment, a pro-inflammatory chemokine well known to promote the differentiation of activated CD4+ T cells into Th1 and Th17 [51], we observed a decrease of Th1-polarized CD4+ T-cell proportion and an increase of Th2 CD4+ T cells. This supports for the first time the impact of collagen hydrolysates on the modulation of CD4 T-cell Th1/Th2 balance in favor of a Th2 activation. Moreover, Naticol^®^Gut treatment also dampens CD8 cytotoxic T-cell activation. These T-cell profiles upon Naticol^®^Gut treatment correlate with an attenuated susceptibility to colitis in Naticol^®^Gut-treated mice. Interestingly, the loss of the capacity of Naticol^®^Gut to decrease the percentage of colonic Th1 CD4 T cells and of activated cytotoxic CD8 T cells upon clodronate administration establishes that Naticol^®^Gut attenuates CD4 and CD8 T-cell inflammatory status through its impact on macrophages. Thus, these data underline the capacity of collagen hydrolysates to control the imbalanced inflammatory immune response in the gut by orienting colonic macrophages toward an immunotolerant phenotype.

In addition to its direct effect on the polarization of macrophages that leads to the attenuation of gut inflammation, Naticol^®^Gut also impacts gut microbiota by promoting protective species such as *Faecalibacterium prausnitzii, Lactobacillus murinus, and Saccharomyces cerevisiae,* well-known probiotic microorganisms that improve intestinal inflammation [45, 52, 53]. In parallel, Naticol^®^Gut limits the development of pathobiontic microorganisms associated with colitis (Enterobacteria as *E. coli, C. albicans*) [44]. Thus, in accordance with the crucial role of microbiota in gut inflammation [54, 55], the reduced severity of colitis by Naticol^®^Gut treatment is dependent on its ability to increase protective bacteria and fungi and to reduce pathobiontic bacteria and fungi burdens in the colon.

It is well known that gut microbiota has immunomodulatory effects by several pathways, such as gut-derived DNA or microbial metabolites, that impact on gut homeostasis [56]. However, there is now growing evidence that, conversely, the immune system also plays a role in shaping the intestinal microbiota composition. Indeed, some immune cell genes play a role in the host gardening of the intestinal microbiome and a disruption of innate immune pathways can alter intestinal microbiota [55, 57]. Our finding showing that the selected bacteria and yeast in vitro growth on minimal media was not changed in the presence of Naticol^®^Gut supports that these collagen hydrolysates have no direct effect on the microorganism development and suggest that the microbiota modulation by Naticol^®^Gut is dependent on its impact on immune system. These observations were in agreement with the fact that collagen peptides are not described as a nutritive matrix for microorganisms or as a surface for biofilm development.

In conclusion, we have identified fish collagen peptides (Naticol^®^Gut) as a protective agent against colitis directly acting on macrophages, by orienting their polarization toward an anti-inflammatory, immunotolerant, and anti-oxidant phenotype in an MR-dependent manner. Moreover, we demonstrated that, through its effect on immune system, Naticol^®^Gut maintains intestinal eubiosis (graphical abstract). Finally, our results on human monocytes from subjects with intestinal inflammation support the use of collagen peptides as new functional food and an innovative and complementary approach in gut health.

## Supplementary Information

Below is the link to the electronic supplementary material.

Supplementary file1 (PDF 672 KB)

Supplementary file2 (PDF 299 KB)
