# Supplementary material for: Bioactive fish collagen peptides weaken intestinal inflammation by orienting colonic macrophages phenotype through mannose receptor activation
Source: Eur J Nutr. 2022 Jan 8;61(4):2051–66. doi: 10.1007/s00394-021-02787-7 (PMC9106617; doi:10.1007/s00394-021-02787-7)
Supplement: Supplementary file 1 — Supplementary file1 (PDF 672 KB) [file 394_2021_2787_MOESM1_ESM.pdf]

Supplementary figure 1

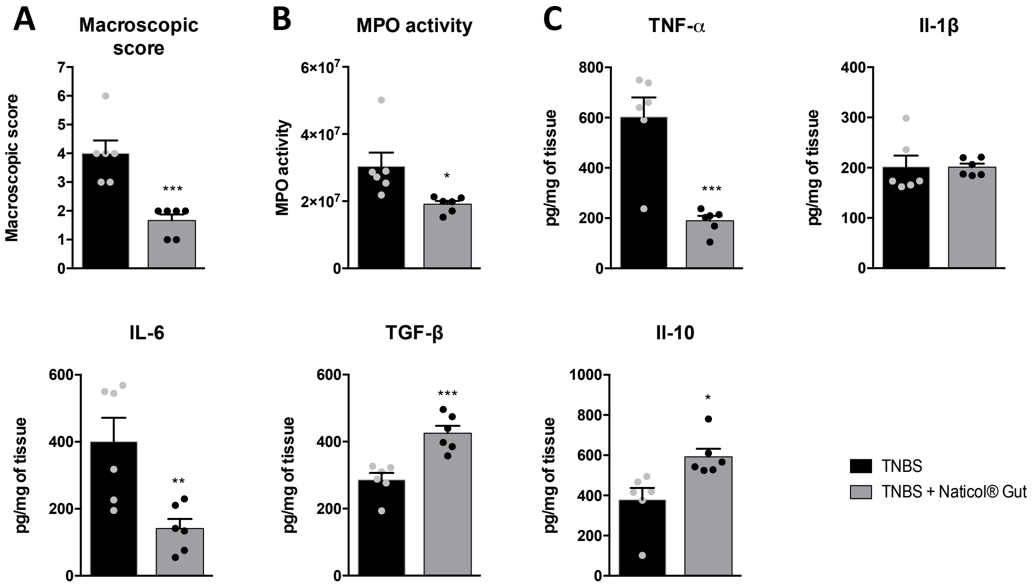

Supplementary Figure 1. Naticol®Gut weakens colonic inflammation in a TNBS-induced colitis
(A) Macroscopic scores, (B) MPO activity, (C) TNF-α, IL-6, IL-1β, TGF-β and IL-10 protein levels were determined 24 hours after TNBS administration in colons of TNBS-exposed mice treated or not with Naticol®Gut (n=6 per group). $^{*}p \leq 0.05$, $^{**}p \leq 0.01$, $^{***}p \leq 0.005$ compared to compared to TNBS exposed-mice.

Supplementary figure 2

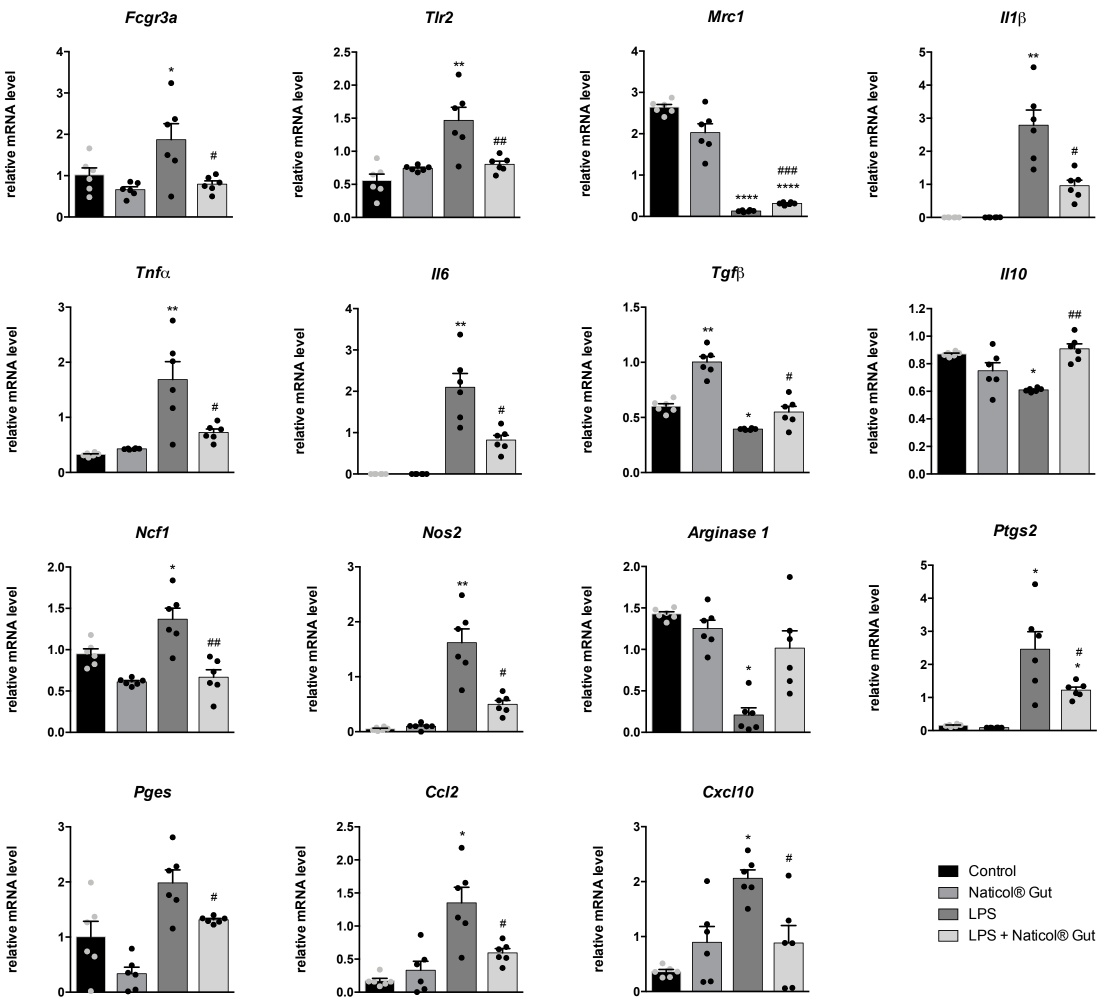

Supplementary Figure 2. Naticol®Gut directly reorients LPS-activated BMDM phenotype towards an anti-inflammatory and anti-oxidant profile. Gene expressio analysis of inflammatory and oxidative stress markers in bone marrow-derived macrophages (BMDM) stimulated or not with LPS and with or without Naticol®Gu using qRT-PCR. Results represent relative mRNA levels. *p≤0.05, **p≤0.01, ****p≤0.001 compared to unstimulated BMDM (control). #p≤0.05, ##p≤0.01, ###p≤0.005 ####p≤0.001 LPS-stimulated BMDM compared to LPS-stimulated BMDM treated with Naticol®Gut.
