# Supplementary material for: Bioactive fish collagen peptides weaken intestinal inflammation by orienting colonic macrophages phenotype through mannose receptor activation
Source: Eur J Nutr. 2022 Jan 8;61(4):2051–66. doi: 10.1007/s00394-021-02787-7 (PMC9106617; doi:10.1007/s00394-021-02787-7)
Supplement: Supplementary file 2 — Supplementary file2 (PDF 299 KB) [file 394_2021_2787_MOESM2_ESM.pdf]

TABLES

Table 1. Amino acid composition of Naticol®Gut (Fish collagen peptides, Weishardt).

Amino acid	Percentage of amino acid
Glycine	20.9
Proline	12.6
Glutamic acid	11.6
Hydroxyproline	10.5
Arginine	8.9
Alanine	8.3
Aspartic acid	5.1
Lysine	3.5
Serine	3.5
Threonine	2.7
Leucine	2.6
Phenylalanine	2.3
Valine	2.0
Isoleucine	1.5
Hydroxylysine	1.5
Histidine	1.3
Methionine	0.8
Tyrosine	0.4
Cysteine+cystine	0.03
Tryptophane	Traces

Table 2. Murine primer sequences used in qPCR assay

Gene	5'- 3'Sequences
Alox15	Sense CAG-GCA-TCG-GAG-TAC-AGG-TT
	Antisense GAT-TGT-GCC-ATC-CTT-CCA-GT
Arginase1	Sense CGT-GTA-CAT-TGG-CTT-GCG-AG
	Antisense TCG-GCC-TTT-TCT-TCC-TTC-CC
Ccl2	Sense AGG-TCC-CTG-TCA-TGC-TTC-TG
	Antisense TCT-GGA-CCC-ATT-CCT-TCT-TG
Ccl22	Sense GTC-CTA-GGG-AGG-AGG-ACC-TG
	Antisense GAA-GGG-GGA-TAA-GCT-GGA-AG
Ccr2	Sense AGA-GAG-CTG-CAG-CAA-AAA-GG
	Antisense GGA-AAG-AGG-CAG-TTG-CAA-AG
Fgcr3a	Sense TGT-TTG-CTT-TTG-CAG-ACA-GG
	Antisense TGC-TCC-ATT-TGA-CAC-CGA-TA
Cd209	Sense GGC-ACG-AAA-GTG-AGG-CAC-AT
	Antisense AGC-TCA-TCT-CCG-CTC-CTA-CCT
Fgcr2a	Sense AGT-CCA-AGC-CTG-TCA-CCA-TC
	Antisense CAG-TTT-TGG-CAG-CTT-CTT-CC
Cxcl9	Sense CTG-GGG-TTA-AAG-GTG-TGT-GC
	Antisense CTC-GTG-GCG-CTG-AAG-ATG-TC
Cxcl10	Sense CTG-CAG-GAT-GAT-GGT-CAA-GC
	Antisense CTG-AGC-TAG-GGA-GGA-CAA-GG
Gapdh	Sense AAC-TTT-GGC-ATT-GTG-GAA-GG
	Antisense ACA-CAT-TGG-GGG-TAG-GAA-CA
*Il1*β	Sense CAA-CCA-ACA-AGT-GAT-ATT-CTC-GAT-G
	Antisense GAT-CCA-CAC-TCT-CCA-GCT-GCA
Il10	Sense CCA-AGC-CTT-ATC-GGA-AAT-GA
	Antisense TTT-TCA-CAG-GGG-AGA-AAT-CG
Il6	Sense GAG-GAT-ACC-ACT-CCC-AAC-AGA-CC
	Antisense AAG-TGC-ATC-ATC-GTT-GTT-CAT-ACA
Inos	Sense TCC-TGG-ACA-TTA-CGA-CCC-CT
	Antisense ACA-AGG-CCT-CCA-ATC-TCT-GC
Mrc1	Sense ATG-CCA-AGT-GGG-AAA-ATC-TG
	Antisense TGT-AGC-AGT-GGC-CTG-CAT-AG
Ncf1	Sense AGT-GAT-GCG-GAG-ACT-TTG-CT
	Antisense ACC-GGA-GTT-ACA-GGC-AAA-TG
Pgds	Sense AGT-GGT-GGA-GGC-CAA-CTA-TG
	Antisense CCA-GCC-CTC-TGA-CTG-ACT-TC
Pges	Sense CCT-AGG-CTT-CAG-CCT-CAC-AC
	Antisense CAG-CCT-ATT-GTT-CAG-CGA-CA
Ptgs2	Sense AGA-AGG-AAA-TGG-CTG-CAG-AA
	Antisense GCT-CGG-CTT-CCA-GTA-TTG-AG
Tgf-β	Sense AGG-TTG-GCA-TTC-CAC-TTC-AC
	Antisense AGG-GGC-CTC-TAA-GAG-CAG-TC
Tlr-2	Sense TGC-TTT-CCT-GCT-GGA-GAT-TT
	Antisense TGT-AAC-GCA-ACA-GCT-TCA-GG
Tnf-α	Sense AGC-CCC-CAG-TCT-GTA-TCC-TT
	Antisense CTC-CCT-TTG-CAG-AAC-TCA-GG

Table 3. Human primer sequences used in qPCR assay

**Gene**	**Sequences 5’- 3’**
18SrRNA	Sense AAA-CGG-CTACCA-CAT-CCA-AG
	Antisense CCT-CCA-ATG-GAT-CCT-CGT-TA
Cd209	Sense TGG-CTA-TAC-CTG-GGG-ACT-TG
	Antisense AGG-AAT-CCA-AGG-GGC-TAA-GA
Ccr2	Sense TGG-CTG-TGT-TTG-CTT-CTG-TC
	Antisense CCC-GAG-TAG-CAG-ATG-ACC-AT
Fcgr3a	Sense TAC-AGC-GTG-GAG-AAG-GA
	Antisense GCA-CCT-GTA-CTC-TCC-ACT
Mrc1	Sense GGC-GGT-GAC-CTC-ACA-AGT-AT
	Antisense ACG-AAG-CCA-TTT-GGT-AAA-CG
Ptgs2	Sense CAT-GTG-AGT-CCC-TGT-GAT-GG
	Antisense GAC-TGC-AGC-AAA-GAC-ATC

Table 4. Sequences used in microbiota PCR assay

Gene/ Phyla/Family/Genus/Specie	Sequences 5'- 3'
B-actin	Sense GGA-CTT-CGA-GCA-AGA-GAT-GG
	Antisense AGC-ACT-GTG-TTG-GCG-TAC-AG
Total bacteria	Sense ACT-CCT-ACG-GGA-GGC-AGC-AG
	Antisense ATT-ACC-GCG-GCT-GCT-GG
Bacteroidetes	Sense GGA-RCA-TGT-GGT-TTA-ATT-CGA-TGA-T
	Antisense AGC-TGA-CGA-CAA-CCA-TGC-AG
Enterobacteria	Sense GTG-CCA-GCM-GCC-GCG-GTA-A
	Antisense GCC-TCA-AGG-GCA-CAA-CCT-CCA-AG
E. coli	Sense GTT-AAT-ACC-TTT-GCT-CAT-TGA
	Antisense ACC-AGG-GTA-TCT-AAT-CCT-GTT
Firmicutes	Sense AGC-TGA-CGA-CAA-CCA-TGC-AC
	Antisense GGA-GYA-TGT-GGT-TTA-ATT-CGA-AGC-A
L. murinus	Sense TCG-AAC-GAA-ACT-TCT-TTA-TCA-CC
	Antisense CGT-TCG-CCA-CTC-AAC-TCT-TT
F.prausnitzii	Sense GAT-GGC-CTC-GCG-TCC-GAT-TAG
	Antisense CCG-AAG-ACC-TTC-TTC-CTC-C
Fungi	Sense CTT-GGT-CAT-TTA-GAG-GAA-GTA-A
	Antisense GCT-GCG-TTC-TTC-ATC-GAT-GC
Candida spp.	Sense TCG-CAT-CGA-TGA-AGA-ACG-CAG-C
	Antisense TCT-TTT-CCT-CCG-CTT-ATT-GAT-ATG-C
C. albicans	Sense ATT-GCT-TGC-GGC-GGT-AAC-GTC-C
	Antisense TCT-TTT-CCT-CCG-CTT-ATT-GAT-ATG-C
S. cerevisiae	Sense AGG-AGT-GCG-GTT-CTT-TG
	Antisense TAC-TTA-CCG-AGG-CAA-GCT-ACA

Table 5. Strain names and references of the *in vitro* cultivated microorganisms

**Strain name**	**Number**
Candida albicans	ATCC-14053
Saccharomyces cerevisiae	DSM-70449
Escherichia coli	DSM-30083
Enterococcus faecalis	DSM-20478
Lactobacillus murinus	DSM-20452
